# Indents

**Published:** 1918-07

**Authors:** 


### INDENTS

**☞ Brief Articles of Technical or Scientific Value will receive notice and comment. Contributions invited.**

---

*New Method of Constructing Continuous Gum Dentures*

Dr. F. H. Nies presented at the National meeting in New York a method of constructing a continuous gum denture by using porcelain crowns that are fitted into platinum cups which are soldered to the platinum plate. The cups are stamped to fit the cervical end of each tooth after it is ground to place. These cups are then soldered to the plate in their proper places, and a pin is soldered into each cup so that it will project into the pin or post hole of the porcelain tooth. The tooth crowns are then removed and the platinum plate is enameled on the exposed surfaces in the usual way, except that no enamel is permitted in the cups as this would interfere with setting the crowns, which is done with cement after the enameling is completed. The advantages claimed are that the porcelain teeth will not be cracked in firing and they will not change color, they can also be easily replaced if accidentally broken. It would seem to us to produce an unusually heavy and clumsy denture, and the technic of construction should be much more complicated. It certainly would be more expensive to make and in cases of close bites almost impracticable. The denture is fully described and well illustrated in the June issue of *The Journal N. D. A.*

*Kidney Infection as a Result of Pyorrhea*

At weekly intervals the patient had temperature elevations reaching 104° F., preceded by chill. These would last one or two days, followed by several days' remission, with only a fraction of a degree rise in temperature. The urine was cloudy, and cloudy urine could be seen coming from the left ureter on cystoscopy. Immediately after removal of the kidney (which had two pelves and two ureters) the temperature fell to 99° F. and a fraction, fluctuating to normal. Some three weeks after this operation, soreness over the right kidney developed, and the temperature shot up to 104° F. The urine became loaded with pus. The teeth of the patient, who for a period of at least a year had been afflicted with pyorrhea alveolaris of moderate severity, were the most patent source of possible infection. They were promptly removed. There was a prompt remission of the fever and pain over the remaining kidney, and the disappearance of pus in the urine. The patient made an uneventful recovery.—F. S. Crockett, in May *Cosmos.*

*Dental Infections* Baude insists that with enlarged glands in the neck, the primary lesion should be sought in the teeth, before incriminating the tubercle bacilli. If there is not already softening, treatment of the teeth generally brings the return to normal of conditions in the glands. In a large proportion of cases the tubercle bacilli locate only secondarily in the inflamed glands. Ordinary inflammation makes the bed for tuberculosis, in both children and adults. He has encountered cases of chronic septicemia from oral sepsis, and urges that the physician should insist on the teeth being put in order as an indispensable element of whatever treatment he is instituting. If the patient can not afford the time or expense for careful dentistry, the tooth should be extracted.—*Paris Medical Journal.*

*Peanuts as Food* The peanut is unusually interesting for several reasons: First, it produces an average of 1,000 pounds of nuts per acre, containing 2,000,000 units of energy and approximately 220 pounds of protein. If the peanuts are properly handled, they will produce about 500 pounds of peanut flour, which contains 44 per cent. of protein and 7.5 per cent. of fat. This peanut flour blends readily with all flour and flour substitutes as well as cornmeals, and is now an official substitute for flour in Florida. When this thousand pounds of peanuts are crushed, they produce 300 pounds of edible oil. In addition, there is a yield per acre of hay, testing 13 per cent. protein, which is a most excellent food for live stock, which gives an additional 130 pounds of protein per acre.

The peanut can be grown all over the South, is one of the surest crops, and can be produced in less time, with less labor, at smaller cost than most of the staple crops. Every farmer in the South knows how to grow peanuts, and the South in 1919 could easily put in 10,000,000 acres. This would produce 3,000,000,000 pounds of fat, which would take care of the fat shortage, and 5,000,000,000 pounds of peanut flour, running 44 per cent. protein and 7.5 per cent. fats.

Allowing four ounces of protein per day for an adult under the most strenuous working conditions, this production could supply all the protein consumed by 24,000,000 adults under the most strenuous conditions for 365 days. This crop can be produced in four months from the time of planting, and the land on which it was planted can be planted with small grains during the other eight months. Without doubt the peanut will produce more food for man and beast in less time, with less labor and less acreage than any other known crop. This is one crop that can be made to take care of both the meat and fat shortage.

We are manufacturers of peanut flour on a commercial scale, and it is being widely used throughout the South.—B. F. Williamson, Gainesville, Fla., in *Journal A. M. A.*, May 11, 1918.

*Granulomas Advantageous* In my studies of acute and chronic inflammatory conditions involving root ends, I am beginning to consider granulomas as one of the most wonderful arrangements for protection in the human body. Thousands of patients that have had their root canals treated and filled under septic conditions and millions of people who for years had dead and putrescent pulps in their teeth should be thankful for this little protection in defending their system against the invasion of bacteria and their toxins.

If nature does so much to protect the body against infection, why worry about breaking the chain of asepsis about root fillings? Why not try to mummify the pulps? Why not fill the canals with antiseptics and germicidal pastes or remove as much of the pulp as can easily be removed and partly fill the canals and then, in the words of a southern dentist "Just trust to God?" But, would it be logical for a man to go on sinning, breaking the moral laws of God, on the principle of trusting in God? In the scientific sense, is it logical for a man to go on violating the God-given laws of nature as observed in the sciences, breaking these laws, obstructing these laws on the same principle of trusting in God? We know that the laws of nature in the healing of the body require certain conditions for their perfect fulfillment and we know that unless we observe the requirements of those laws, that if we obstruct them with means that are known from our scientific studies to interfere with the normal course of those laws, that an unfair burden is placed on nature's shoulders. Therefore, it is our duty to assist nature, the great doctor, in curing disease by ways and means that are based on the fundamental principles of modern therapy and surgery.

It must be conceded, and I believe it is, that the healing of morbid conditions involving the periapical tissues is not always successful when confining the treatment to root canals; especially is this true in chronic conditions. Root amputation and curettage with proper post-operative treatment is a safe method by which to eliminate a chronic condition, such as root granuloma, epithelial root tumors, root cysts, apical necrosis, rarefying ostitis, exostosis. It is also indicated in root ends that can not be filled to the very end. —Dr. Federspiel, in *Dental Items of Interest.*

*War Substitutes* Economy for Waste.
Cooperation for Criticism.
Knowledge of Prices for Gossip about Profits.
Cornmeal and Oatmeal for Wheat Flour.
Fish for Beef and Bacon.
Vegetable Oils for Animal Fats.
The Garden Hoe for the Golf Stick.
Performance for Argument.
Service for Sneers.
Patriotic Push for Peevish Puerilities.
Perishable for Preservable Foods.
Greater Production for a German Peace.
The Beef You Do Not Eat for the Rifle You Can Not Carry.
Conservation for Conversation.
Common Sense for Common Gossip.
Marketing for Telephoning.
Production for Pessimism.—*Canadian Food Bulletin.*

*Saving in Concrete Terms* Another economic theme which the Food Administration has had to pioneer is that of saving. Speaking broadly, we have some 36,000,000 of able-bodied manhood. We have already had to divert 2,000,000 of these men to actual arms. Beyond this, we have had to divert a vast number of men to provide munitions not only for ourselves but for the allies. We have had to divert vast numbers of men to the provision of the raw materials for these shops. We have had to set aside larger amounts of our foodstuff for the allies, and consequently there was a diversion of farm production to this purpose.

Altogether a rough calculation indicates that already we have diverted from eight to ten million men from their normal occupations toward war and the products it requires. That is from one-quarter to one-third of our normal productive units. It is possible that we can increase the exertion of the remainder of our productive population by eliminating nonessential labor by more intensive labor and longer hours, by the application of woman's labor, by putting the boys into labor earlier than otherwise, and can make up some of the gap in our productive units. We can not, however, compass the whole, and the deficiency can only be overcome by the reduction in the consumption of commodities.

This does not apply to food alone; it applies to every commodity of which we consume more than is necessary for our health and comfort. We must strip to the bone in order that we may afford the economic luxury of the diversion of this portion of our productive power to the destruction of war. If we do not, our exertion in this war will stop short of the task imposed upon us, and we can not look to victory with any assurance.—H. Hoover.

*To Renew Arkansas Stones* When the surface of your Arkansas stone has become oil soaked and worn unevenly, it may be resurfaced as good as new by rubbing back and forth with a planing motion upon a piece of emery cloth laid "face up" upon the surface of the laboratory bench.—P. G. Puterbaugh, in *Review.*

*Sterilization of the Dental Office* It is undoubtedly true that our dental offices, as offices, are poorly arranged for the purpose. We have too many hangings, too many oriental rugs, too many domestic rugs, too many dust-catchers, too many dirty corners, too much rubbish lying around, and we are not careful enough in other ways. The only way of correcting my own errors would be to throw all the equipment I have in the scrap heap, rent a new office and begin at the foundation, for I want an office that can be cleaned. I want an office which I am not dependent upon the janitor of the block to come in and clean for me.

We have gone at the question of sterilization in a very haphazard way. We have been buying a lot of nice, expensive, white-enamel furniture, and putting that up to catch the eye, to please the public, and lead them to think that we are being aseptic in our operations. Now, we are not aseptic in any sense of the word. I know I am not, and can not be until I have a different office.

The matter of the sterilization of every instrument used for a patient before it is used for another case is absolutely essential, and can be practiced by anyone. Better care of the hands and of the materials we use is necessary. The ordinary paper points, while they may be sterile when made, are not sterile when we get them. We may sterilize our instruments in the sterilizer, and believe that they are sterile when they are taken from the apparatus. We bring them into the operating room, put them in a case, the dust settles on them, and we no longer have sterile instruments.—Dr. Cook, in *Cosmos.*

*How Potato Flour is Made*

The pioneer marketer of the old Oregon trail, Ezra Meeker, is not satisfied with the laurels he obtained in the earlier days and he is now again serving his country by pioneering in the manufacture of potato flour.

Potato flour is not a new product to Mr. Meeker, for he manufactured this product for the Alaska trade years ago. Mr. Meeker recently stated that a plant for handling potatoes can be constructed for a few hundred dollars from material obtainable anywhere in his section, which is in the extreme northwest.

"A ton of potatoes," says Mr. Meeker, "will produce 500 pounds of flour." The process he describes is simple and inexpensive. First the potatoes are washed clean and then sliced with the peelings on and dropped immediately into clean water to prevent discoloration and to rinse them; then as soon as practicable they are either parboiled or steamed from 8 to 10 minutes—long enough to cook the starch—when the slices lose their opaque appearance and become transparent. The cooked slices are then transferred to a drier and for the first few hours subjected to a current of hot air not greater than 120° F., after which the temperature is gradually increased to 170°, but no greater.

The drying process is continued until the slices are brittle, though it is immaterial if a few here and there are not thoroughly dried. When taken off the kiln, the dried product is placed in a compact pile in dry room and handled over daily for three or four days until the pile "evens up," after which they are ready for the mill to grind them into potato meal or flour.

A revolving washer—a long box partially emerged in a pan of water—can be cheaply built to easily wash a ton of potatoes an hour. An ordinary root cutter, which may be purchased for $30 or less, will answer to slice the potatoes, but it is likely that a more desirable machine for slicing may be found on the market. The average mill will cost from $150 to $225 and up, but at present can not be obtained on short notice.

Mr. Meeker mentions one potato-flour plant at Yakima, Wash., that is producing 25 to 30 barrels of flour a day. In April this flour was selling for $21 a barrel.

*One Non-Essential Industry*

The question as to what constitutes a non-essential industry in war time can not be answered offhand. The complexities of modern business are such as to make it extremely difficult to determine just what industries should be suspended under the stress of war conditions without inflicting a greater economic injury than is justified by the possible benefits that might accrue. There is one business, however, that those who have given the problem any study would have to admit should be largely dispensed with during the war—and might properly be largely dispensed with at all times—and that is the manufacture of nostrums. Every physician and every druggist knows that there is not a "patent medicine" on the market today whose place is not better filed by official products that are found on the shelves of every druggist in the country. Every physician and every druggist knows that the "patent medicine" maker aims, not to furnish drug products that can be obtained in no other form, but to duplicate in infinite variety, with secret mixtures, the non-secret preparations already on the druggists' shelves and, what is far worse, to stimulate unnecessary self-drugging on the part of the public. It is true that, if the "patent medicine" industry were destroyed, druggists would for awhile suffer financial loss, not because they derive a large profit from "patent medicines," but because the public, being deprived of the lying scare-heads of "patent medicine" advertisements, would buy fewer drugs of every kind. Certainly, however, this loss to some thousands of druggists would be an immeasurable gain to some millions of people. The exploiters of a widely heralded nostrum have bragged in their advertisements of the number of carloads of their mischievous alcoholic mixture that they have shipped to certain points. Does even the intelligent layman believe that under present conditions there is any excuse for taking up valuable shipping space with such worthless, if not dangerous, trash? And this is but one of scores. If there is one industry in the United States whose suspension at the present time is justified on the grounds of public health, greater efficiency and avoidance of waste, it is the nostrum industry.—*Journal A. M. A.*, May 25.

*Dental Treatment for Mothers*

Dr. J. Francis Taylor, the medical officer of health for Leyton, reports that the infant welfare and ante-natal clinics are making rapid strides in popularity. Attendances and results are most satisfactory, but he suggests that in order to develop their usefulness an extension is advisable in the direction of providing dental treatment for actual and prospective mothers. It is found that the health of the mothers is seriously prejudiced by the presence of decaying teeth, which act as a septic focus from which toxins are absorbed into the blood, and these have been proved to affect injuriously the unborn infant and also the milk of the nursing mother. There is already a dental clinic for school children, with a dentist and nurse conducting the work, and he suggests that arrangements should be made for two sessions at the ante-natal clinic. Dr. Taylor would administer the necessary anesthetics, as he does in the case of the school children. The total expenditure, excepting a small sum for fillings, would not exceed £125 per annum, one-half of which would be payable by the Local Government Board. Dr. Taylor predicts that incalculable benefit would be conferred on the mothers and infants by this modest outlay. The Health Committee has agreed to this proposal.—British *Dental Record.*

*Aniline Dye as a Germicide* The following method of preparation and application has been used with much success in my practice for the past year, in treating chronic abscess and pyorrhea. Make a solution in the proportion of about half a grain of aniline violet (Methol Violet B.) to one drachm of distilled water. Inject a small quantity of the solution into the fistulous opening of the abscess, with a blunt pointed syringe. The syringe should have a glass barrel, and the point either gold or platinum. The abscess should be treated surgically first. If necessary it should be curetted or in a case of root excision the parts should first be properly cared for. The aniline solution should be used three or four times at intervals of every other day. It may be applied with cotton twisted on a broach. In severe chronic cases or in pyorrhea pockets a small pledget of cotton may be saturated and pressed into the orifice and left for twenty-four hours. The aniline treatment may be followed with bismuth paste as a final dressing. In using aniline dye it is necessary to use some care only on account of the color, as there is absolutely no danger because of the stain on lips or tongue. Besides the stain can readily be removed with absorbent cotton and water.—Geo. D. Sitherwood, in *Dental Review.*

